# Observation of microwave absorption and emission from incoherent electron tunneling through a normal-metal–insulator–superconductor junction

**DOI:** 10.1038/s41598-018-21772-5

**Published:** 2018-03-02

**Authors:** Shumpei Masuda, Kuan Y. Tan, Matti Partanen, Russell E. Lake, Joonas Govenius, Matti Silveri, Hermann Grabert, Mikko Möttönen

**Affiliations:** 1 QCD Labs, QTF Centre of Excellence, Department of Applied Physics, Aalto University, PO Box 13500, AALTO, FI-00076 Finland; 2 Research Unit of Theoretical Physics, University of Oulu, Oulu, FI-90014 Finland; 3 Department of Physics, University of Freiburg, Freiburg im Breisgau, Germany

## Abstract

We experimentally study nanoscale normal-metal–insulator–superconductor junctions coupled to a superconducting microwave resonator. We observe that bias-voltage-controllable single-electron tunneling through the junctions gives rise to a direct conversion between the electrostatic energy and that of microwave photons. The measured power spectral density of the microwave radiation emitted by the resonator exceeds at high bias voltages that of an equivalent single-mode radiation source at 2.5 K although the phonon and electron reservoirs are at subkelvin temperatures. Measurements of the generated power quantitatively agree with a theoretical model in a wide range of bias voltages. Thus, we have developed a microwave source which is compatible with low-temperature electronics and offers convenient *in-situ* electrical control of the incoherent photon emission rate with a predetermined frequency, without relying on intrinsic voltage fluctuations of heated normal-metal components or suffering from unwanted losses in room temperature cables. Importantly, our observation of negative generated power at relatively low bias voltages provides a novel type of verification of the working principles of the recently discovered quantum-circuit refrigerator.

## Introduction

Superconducting circuits provide a promising platform for quantum technological applications^1^, such as quantum information processing^2–8^, sensing^9–11^, and refrigeration of electric components^12–15^. In particular, microwave photons constitute a fundamental and controllable medium^16^ for energy and information transport between quantum electric components. For example, quantum-limited heat conduction mediated by microwave photons has been measured^13,17^, even through coplanar waveguides (CPW) across macroscopic distances^18^.

In this framework, both incoherent and coherent microwave sources on the same chip with their target are highly desirable since they minimize unwanted and difficult-to-calibrate losses which typically occur in microwave cables and connectors. Such an incoherent source with high dynamic range can provide reference power with a given frequency for calibration of cryogenic devices^19^ such as microwave photon detectors^10^ and for experiments on photon statistics^20^, thermal transport^15^, and quantum-limited amplifiers^21^. A coherent on-chip source^22,23^ on the other hand, can potentially be used to drive qubits in the emerging large-scale quantum computers without detrimental heat loads from microwave lines between different temperature stages of the cryostat. Thus, on-chip creation of microwave photons^24–26^ and improvement of the dynamic range of the sources are of great interest to electric quantum circuits.

It is well known that tunneling of electrons across a nanoscale barrier can lead to simultaneous energy exchange with the coupled electromagnetic environment such as a resonator^14,15,19,27–39^. Consequently, several schemes for exciting the resonator based on this kind of photon-assisted electron tunneling have been introduced using, for example, Josephson junctions^22,23,40,41^ and quantum dots^42–45^ (see also^46–49^ for schemes utilizing superconducting qubits). In contrast, refrigeration of a superconducting microwave resonator mode has been recently demonstrated using voltage-controlled tunneling of single electrons across normal-metal–insulator–superconductor (NIS) tunnel junctions^14^. To the best of our knowledge, however, no microwave source based on metallic NIS tunnel junctions has been demonstrated to date.

In this paper, we report the observation of microwave absorption and emission arising from photon-assisted single-electron tunneling through NIS junctions. The experimental setup is extended from that of the first quantum-circuit refrigerator^14^, such that we can measure the power spectral density of the microwave radiation generated in a CPW resonator. The device is compatible with low-temperature electronics and offers orders of magnitude electrical tunability of the generated power with the frequency corresponding to the lowest mode of the CPW resonator. Importantly, we observe that the resonator mode is cooled by the quantum-circuit refrigerator by measuring for the first time the effect of the refrigerator on the emitted radiation. Furthermore, our results demonstrate that the mode temperature of the resonator can be driven beyond 2.5 K, far above the temperatures of the phonon and electron reservoirs of the system. Thus when operated as an incoherent microwave source, our device has potential in delivering relatively high powers without the excess heating of other nearby components or sensitivity to modest external magnetic fields.

## Results and Discussion

**Figure 1:**
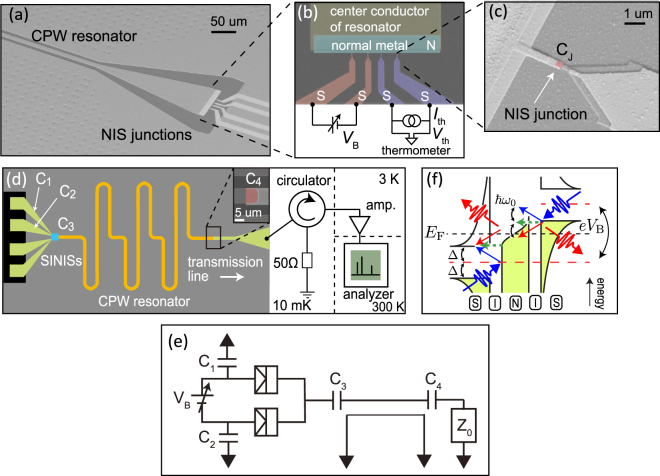
(**a**) Scanning-electron microscope (SEM) image of a fabricated device illustrating the CPW resonator and the NIS junctions operating together as the microwave source. (**b**) Colored SEM image of the NIS junctions. The leads highlighted in red are used to excite the resonator using the bias voltage *V*_B_. The leads highlighted in blue are used as a thermometer to measure the electron temperature of the normal-metal island. (**c**) SEM image of an NIS junction highlighted in red, where *C*_J_ denotes the junction capacitance. (**d**) Device design and measurement scheme. Here, *C*_1–2_, *C*_3_, and *C*_4_ denote the coupling capacitances between the bonding pads and the ground plane, between the normal-metal island of the NIS junctions and the center conductor of the resonator, and between the resonator and the transmission line (see inset), respectively. The area indicated by the blue square corresponds to panel (a) although the axis of view is different. (**e**) Simplified circuit diagram showing the connections of the labeled components in (d). Here, ${Z}_{0}\simeq 50$ Ω is the characteristic impedance of the transmission line. (**f**) Energy diagram for single-electron tunneling at bias voltage *eV*_B_/(2Δ) > 1, where & planck;*ω*_0_ is the energy of an emitted or absorbed photon. The black solid curves at the normal metal and the superconductors represent the Fermi–Dirac distribution function and the density of states in the superconductors, respectively. The colored areas represent the occupied states. The straight arrow represents the electron tunneling from the initial energy state (beginning of the arrow) to a final energy state (end of the arrow). The wavy arrows denote absorbed or emitted photons in the tunneling process.

The experimental sample shown in Fig. 1(a)–(c) consists of NIS tunnel junctions capacitively coupled to a half-wave-length superconducting CPW resonator fabricated on a high-purity 500-*μ*m-thick silicon wafer. The silicon substrate is passivated with a 300-nm-thick thermally grown silicon dioxide. The resonator is defined with photolithography and reactive ion etching of a 200-nm-thick sputtered Nb layer. Subsequently, a 50-nm-thick layer of Al_2_O_3_ is introduced on the whole wafer using atomic layer deposition (ALD) process at 200 °C. This ALD oxide serves as the dielectric material of the parallel plate capacitors *C*_1–4_ shown in Fig. 1(d). The NIS junctions and a transmission line connected to the resonator are subsequently defined using electron beam lithography, followed by a standard two-angle evaporation with *in-situ* oxidation to form the tunnel barriers. The final NIS nano-structure consists of a 20-nm-thick normal metal (Cu) on top of a 20-nm-thick superconductor (Al), separated by a thin aluminum-oxide tunnel barrier. The tunnel junction area is approximately 200 × 200 nm^2^ inferred from the SEM image of the sample.

Figure 1(d) depicts the layout of the sample and the measurement setup with a simplified circuit diagram given in Fig. 1(e) (see Sec. III of Supplementary information for more details). Microwave photons generated in the resonator at 10-mK cryostat temperature decay into the transmission line, and are characterized using a spectrum analyzer. As depicted in Fig. 1(b), a pair of voltage-biased NIS junctions is used to create the photons, while a current-biased pair is used as a thermometer to measure the electron temperature of the normal-metal island^50^ (see Sec. V of Supplementary information). To estimate the power generated by controlled electron tunneling without offsets, we subtract the measured power corresponding to the off-state of the source, namely, zero bias voltage, from the power measured at finite bias voltages. We refer to this quantity as the generated power.

To enhance the interaction between the tunneling electrons and the resonator, we use a large coupling capacitance between the normal-metal island and the resonator, *C*_3_, in comparison to the tunnel junction capacitance, *C*_J_. For the sake of convenient experimental detection, the coupling capacitance between the resonator and the transmission line is chosen to maintain a well-defined resonance although with a relatively low loaded resonator quality factor *Q* ≈ 60. Note that the source may also be conveniently designed for narrow-bandwidth operation. The resonator length is chosen to obtain the desired frequency of the generated radiation. In this work, we examine two samples with resonator lengths of *L*_res_ = 13.14 mm (Sample A) and 6.89 mm (Sample B) which correspond to the fundamental resonance frequencies of 4.55 GHz and 8.32 GHz, respectively.

Although elastic tunneling, where the electron does not exchange energy with the resonator, is typically frequent in our experiments, photon-assisted electron tunneling is the key phenomenon leading to the photon creation we observe below. When an electron tunnels across an NIS junction, it can absorb energy from or emit energy to the resonator, that is, annihilate or create photons in the resonator^30^. Figure 1(f) illustrates elastic and photon-assisted single-electron tunneling events across the junctions and the associated photon creation and annihilation. If the energy provided by the bias voltage to the electron tunneling across the junction, *eV*_B_/2, is greater than the superconductor gap parameter, Δ, the photon creation rate increases faster with the bias voltage than the annihilation rate. Thus, the effective temperature related to the creation and annihilation of photons increases. The elastic tunneling does not directly affect the resonator modes but typically dominates the electric current across the junctions for $e{V}_{{\rm{B}}}\mathrm{/2}\gtrsim {\rm{\Delta }}$.

**Figure 2:**
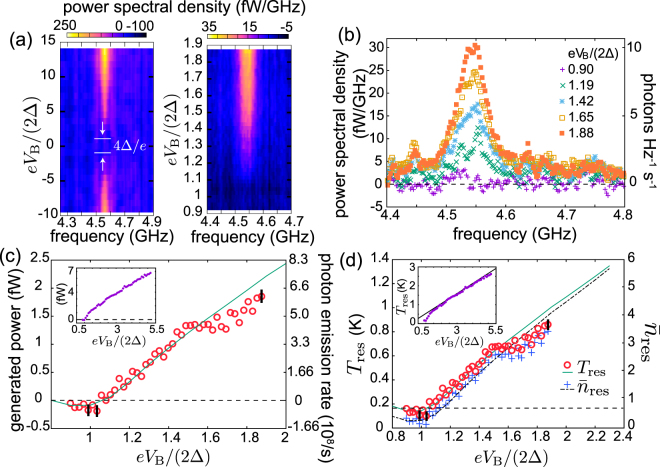
Experimental and numerical results for Sample A with the fundamental resonance frequency *f*_0_ = 4.55 GHz. (**a**) Spectral densities of the generated power measured as functions of frequency and bias voltage. Each frequency trace is measured by a spectrum analyzer as shown schematically in Fig. 1(d) and averaged over 21000 repetitions. In addition, we employ a three-point moving average in frequency. The left panel shows the generated power spectral density in a wider voltage range compared with the right panel. In contrast to Fig. 1(d), no circulator was used for the data in the left panel. (**b**) Power spectral density and the corresponding density of photon emission rate (photons Hz^−1^ s^−1^) of (a) as functions of frequency for the indicated bias voltages. The horizontal dashed line indicates the zero level corresponding to zero bias voltage. (**c**) Generated power, obtained by integrating data as in (b) from 4.4 GHz to 4.8 GHz. The green curve indicates the theoretical prediction of the thermal model illustrated in Fig. 3. The inset shows the generated power for an extended bias voltage range. The dashed lines indicate the zero level. (**d**) Average photon number and temperature of the fundamental mode as functions of the bias voltage obtained using the experimental results in (c) and Eqs (2) and (3). The parameters are given in Table 1. Here, the dashed and solid curves represent ${\bar{n}}_{{\rm{res}}}$ and *T*_res_ obtained from the thermal model, respectively. Representative 1*σ* uncertainties of the measured data points are shown by black vertical lines in (c) and (d). The solid line in the inset of (d) represents ${T}_{{\rm{r}}es}=e{V}_{{\rm{B}}}\mathrm{/(4}{k}_{{\rm{B}}}+2{k}_{{\rm{B}}}{R}_{{\rm{T}}}{Z}_{0}{C}_{4}^{2}{\omega }_{0}^{2})$, which is obtained in the high voltage regime where ${\bar{n}}_{{\rm{r}}{\rm{e}}{\rm{s}}}\approx {k}_{{\rm{B}}}{T}_{{\rm{r}}{\rm{e}}{\rm{s}}}/(\hslash {\omega }_{0})$^51^. We used a 38.5-dB cryogenic amplifier, 20-dB room temperature amplifier, and 6-dB room temperature attenuator. We assumed 11.9 dB of loss from the PCB and rf cables as opposed to 7.5 dB calibrated with a transmission measurement of a control sample. We attribute the discrepancy in the loss mainly to the fact that the microwaves propagate through additional cables in the calibration measurement, which leads to excess uncertainties. The horizontal dashed line indicates the apparent temperature of the transmission line as given in Table 1.

Figure 2(a) and (b) show the spectral density of the generated power as a function of the bias voltage for Sample A. We observe increased generated power around 4.55 GHz matching the frequency of the fundamental resonator mode estimated using the experimental parameters given in Table 1. The spectral brightening can be mostly described by a characteristic Lorenzian peak. We attribute the additional structure to microwave reflections from the circuitry following the resonator.

**Table 1:** Parameters of the experimental samples (see Sec. II of Supplementary information for details): the length of the resonator *L*_res_, superconductor gap parameter Δ, Dynes parameter *γ*_D_, capacitance per unit length *c*_res_, inductance per unit length *l*_res_, fundamental resonance frequency *f*_0_, tunnel resistance *R*_T_, junction capacitance *C*_J_, capacitance *C*_3/4_, characteristic impedance of the transmission line *Z*_0_, apparent temperature of transmission line *T*_TL_, the employed attenuation of the PCB and rf cables *α*, the width of the center conductor *w*, and the separation between the center conductor and the ground plane of the CPW resonator *s*. Capacitance *C*_1/2_ is more than 50 times larger than *C*_3_.

	SAMPLE A	SAMPLE B		SAMPLE A	SAMPLE B
*L*_res_	13.14 mm	6.89 mm	*C*_3_	0.84 pF	0.78 pF
Δ	220 *μ*eV	191 *μ*eV	*C*_4_	0.072 pF	0.079 pF
*γ*_D_	4 × 10^−4^	4 × 10^−4^	*Z*_0_	53 Ω	52 Ω
*c*_res_	159 pF/m	169 pF/m	*T*_TL_	180 mK	150 mK
*l*_res_	0.45 *μ*H/m	0.45 *μ*H/m	*α*	11.9 dB^a^	17 dB^b^
*f*_0_	4.55 GHz	8.32 GHz	*w*	7.8 *μ*m	8.0 *μ*m
*R*_T_	12.5 k Ω	10.0 kΩ	*s*	5.7 *μ*m	6.1 *μ*m
*C*_J_	2 fF	2 fF			

^a,b^The values experimentally measured with a control sample are 7.5 dB^a^ and 13 dB^b^.

The power spectral density increases with the bias voltage for |*eV*_B_/(2Δ)| > 1 whereas it is almost vanishing for |*eV*_B_/(2Δ)| < 1. This onset of generated radiation matching the energy gap in the superconductor density of states provides clear evidence that single-electron tunneling is responsible for the observed photon emission. In the units of the photon emission rate per hertz, the observed maximum power spectral density of roughly 80 Hz^−1^s^−1^ at $e{V}_{{\rm{B}}}\mathrm{/(2}{\rm{\Delta }})\simeq 14$ is orders of magnitude higher than those reported in refs^41,43^ for incoherent sources. Some coherent sources^22,23^ provide yet orders of magnitude higher power spectral density. However, our incoherent source is based on a different physical phenomenon. Thus, its output power has a simple bias-voltage dependence, offering robust *in-situ* electrical control. The bias-voltage dependence of the frequency-integrated spectral density, i.e., the generated power is shown in Fig. 2(c). The onset of positive generated power is clearly visible at $e{V}_{{\rm{B}}}\mathrm{/(2}{\rm{\Delta }})\gtrsim 1$.

Owing to energy conservation, the net power flowing from the resonator to the transmission line is given by *P*_RT_ = *P*_out_ − *P*_in_, where *P*_out_ and *P*_in_ are the powers carried by photons moving in the transmission line away from and towards the microwave source, respectively. The net power is related to the average photon number of the resonator, ${\bar{n}}_{{\rm{res}}}$, and of the radiation incident on the resonator from the transmission line, ${\bar{n}}_{{\rm{TL}}}$, at the fundamental angular frequency, *ω*_0_, by (see Sec. IV of Supplementary information)

1 $${P}_{{\rm{RT}}}=\frac{2{C}_{4}^{2}\hslash {\omega }_{0}^{3}{Z}_{0}}{{L}_{{\rm{res}}}{c}_{{\rm{res}}}}({\bar{n}}_{{\rm{res}}}-{\bar{n}}_{{\rm{T}}{\rm{L}}}),$$

where *Z*_0_ is the characteristic impedance of the transmission line and *c*_res_ is the capacitance per unit length of the resonator. The circulator shown in Fig. 1(d) renders *P*_in_ and ${\bar{n}}_{{\rm{TL}}}$ independent of the bias voltage, and hence the generated power assumes the form

2 $${\rm{\Delta }}{P}_{{\rm{out}}}:={P}_{{\rm{out}}}({V}_{{\rm{B}}})-{P}_{{\rm{out}}}\mathrm{(0)}=\frac{2{C}_{4}^{2}\hslash {\omega }_{0}^{3}{Z}_{0}}{{L}_{{\rm{res}}}{c}_{{\rm{res}}}}[{\bar{n}}_{{\rm{res}}}({V}_{{\rm{B}}})-{\bar{n}}_{{\rm{res}}}\mathrm{(0)].}$$

Although the above equation only provides a direct conversion from the measured generated power to the change of the average resonator photon number, we describe in Sec. IV of Supplementary information how we extract the photon number at zero bias with the help of our theoretical model. In the model, we assume for simplicity that the exchanged power *P*_RT_ vanishes at zero bias voltage, i.e., here the resonator is thermalized with the tranmission line. This assumption does not lead to artificial changes in the estimated ${\bar{n}}_{{\rm{res}}}$, but renders ${\bar{n}}_{{\rm{TL}}}$ to describe the joint voltage-bias-independent heating effect of the resonator owing to the transmission line and any additional environments. Consequently, we refer below to *T*_TL_ as the apparent transmission line temperature. Note that the estimated resonator temperature becomes insensitive to changes in *T*_TL_ at high bias voltages where ${\bar{n}}_{{\rm{res}}}\gg {\bar{n}}_{{\rm{TL}}}$. In our experiments, the resonator mode is in a thermal state described by temperature *T*_res_, and hence we may describe its average photon numbers using the Bose distribution function as

3 $${\bar{n}}_{{\rm{res}}}=\frac{1}{\exp [\hslash {\omega }_{0}/({k}_{{\rm{B}}}{T}_{{\rm{res}}})]-1}\mathrm{.}$$

Figure 2(d) shows the resonator temperature and average photon number corresponding to the measured generated power in Fig. 2(c) as functions of the bias voltage. These results indicate that the mode temperature and the average photon number can be efficiently controlled using the bias voltage. For *eV*_B_ > 10Δ, we have *T*_res_ > 2.5 K. Such a high mode temperature is not conveniently achieved by coupling the resonator to a hot resistor due to the transition of the superconducting aluminum, employed as the lead material, to the normal state.

Importantly, the measured electron temperature shown in Sec. V of Supplementary information is much lower than that of the resonator mode for almost any bias voltage in Fig. 2(d). This observation verifies that the observed microwave radiation directly arises from photon-assisted electron tunneling rather than from heating of the normal-metal island.

Although negative generated power is challenging to differentiate in the measured power spectra, we observe in Fig. 2(c) a shallow but statistically significant dip in the experimental generated power around *eV*_B_/(2Δ) = 1. The dip corresponds to the refrigeration of the fundamental mode owing to photon-assisted tunneling, and hence provides complementary evidence of this phenomenon first observed in ref.^14^. As theoretically shown in refs^14,51^, the photon-assisted tunneling acts on the resonator as an environment, the effective temperature of which achieves a minimum of half the electron temperature somewhat below the gap voltage. This qualitatively explains the observed refrigeration. At high bias voltages, the effective temperature approaches *eV*_B_/(2*k*_B_)^51^, and hence a local minimum in the generated power appears.

**Figure 3:**
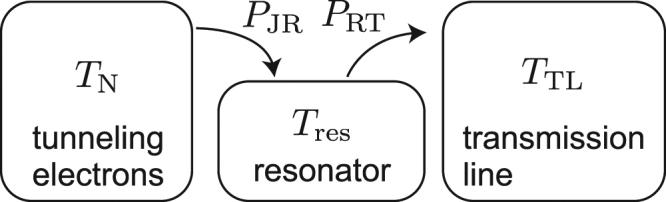
Thermal model of the device indicating the relevant temperatures and energy flows. The temperature of the fundamental resonator mode, *T*_res_, becomes higher than the apparent temperature of the transmission line, *T*_TL_, when the applied bias voltage strongly drives photon creation. Here, *T*_N_ is the electron temperature at the normal-metal island of the NIS junctions. At steady state, the power *P*_JR_ from the tunneling electrons to the resonator and the power *P*_RT_ from the resonator to the transmission line balance, *P*_JR_ = *P*_RT_. See Sec. III and IV of Supplementary information for details on modeling the power flows.

To describe the energy transfer from the tunneling electrons to the transmission line, mediated by microwave photons in the fundamental mode of the resonator, we develop a thermal model shown in Fig. 3. Here, we consider thermal states for the electric components assuming that the temperatures of the resonator mode, *T*_res_, and of the electrons in the normal-metal island, *T*_N_, are well defined in the parameter range studied. We have verified the validity of this assumption with a model that accounts for transitions between individual resonator states^51^ but do not present the model here since its predictions are essentially identical to those provided by our simpler model. Because of the relatively weak coupling between the resonator and the transmission line, we assume that a tunneling electron does not induce photon-assisted transitions in the transmission line. Instead, the influence of the tunneling events is indirectly taken into account through the change in the temperature of the resonator mode.

In the thermal model, the power from the tunneling electrons to the resonator, *P*_JR_, is calculated using the *P*(*E*) theory^30^, as detailed in Sec. III of Supplementary information. Both powers, *P*_JR_ and *P*_RT_, are functions of the temperature of the resonator mode. We solve *P*_RT_ for each *V*_B_ by finding the mode temperature, at which these powers balance, *P*_JR_ = *P*_RT_. Subsequently, the average photon number ${\bar{n}}_{{\rm{res}}}$ is calculated using the obtained mode temperature and Eq. (3). We have measured the bare internal quality factor of samples with the same design to be orders of magnitude higher than the realized external quality factor and hence we neglect the effect of any additional internal losses to the power balance. The apparent temperature of the transmission line, corresponding to the photons moving towards the resonator and possible additional heating channels, is assumed to be independent of the applied bias voltage. The experimentally measured electron temperature of the normal-metal island is used in the simulation.

The theoretical results, employing the parameters given in Table 1, show in Fig. 2(c) good agreement with the experiments in a wide range of generated powers. In the model, the dip in the generated power around *eV*_B_/(2Δ) = 1 is arising from photon absorption induced by electron tunneling. Furthermore, the modelled average number of photons, ${\bar{n}}_{{\rm{res}}}$, and the temperature of the fundamental mode, *T*_res_, are shown in Fig. 2(d) as functions of the bias voltage.

**Figure 4:**
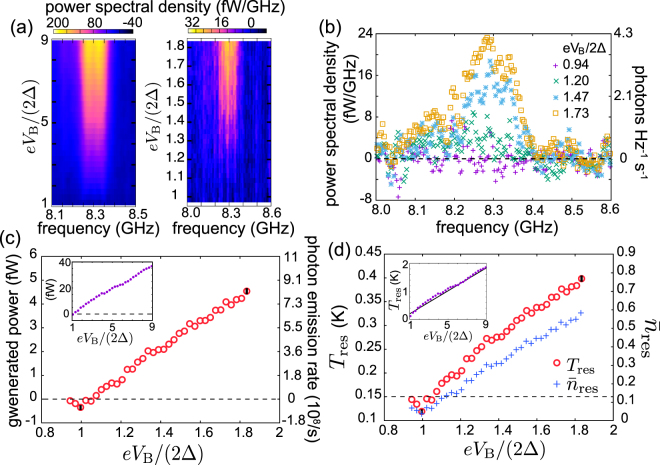
Experimental results for Sample B with the fundamental resonance frequency *f*_0_ = 8.3 GHz. (**a**) Spectral densities of the generated power measured as functions of frequency and bias voltage. The left panel shows the generated power spectral density in a wider voltage range compared with the right panel. (b) Power spectral density and the corresponding density of photon emission rate (photons Hz^−1^ s^−1^) of (a) as functions of frequency for the indicated bias voltages. The horizontal dashed line indicates the zero level. (**c**) Generated power, obtained by integrating data as in (b) from 8.0 GHz to 8.6 GHz. The inset shows the generated power for an extended bias voltage range. The dashed lines indicate the zero levels. (**d**) Average photon number and temperature of the fundamental mode as functions of the bias voltage obtained using the data in (c) and Eqs (2) and (3). The parameters are given in Table 1. Representative 1*σ* uncertainties of the measured data points are shown by black vertical lines in (c) and (d). The solid line in the inset of (d) represents ${T}_{{\rm{res}}}=e{V}_{{\rm{B}}}\mathrm{/(4}{k}_{{\rm{B}}}+2{k}_{{\rm{B}}}{R}_{{\rm{T}}}{Z}_{0}{C}_{4}^{2}{\omega }_{0}^{2})$, which is obtained in the high voltage regime where ${\bar{n}}_{{\rm{r}}{\rm{e}}{\rm{s}}}\approx {k}_{{\rm{B}}}{T}_{{\rm{r}}{\rm{e}}{\rm{s}}}/(\hslash {\omega }_{0})$^51^. We used a 40-dB cryogenic amplifier, 20-dB room temperature amplifier, and 6-dB room temperature attenuator. We assumed 17 dB of loss from the PCB and rf cables as opposed to 13 dB measured using a control sample. The horizontal dashed line indicates the apparent temperature of the transmission line as given in Table 1. In contrast to Fig. 2(c) and (d), we do not show theoretical results here due to the lack of reliable temperature data for the normal-metal electrons.

Experimental results similar to those discussed above are shown in Fig. 4 for Sample B. Figure 4(a) shows the spectral density of the generated power as a function of the frequency and the bias voltage. A peak appears around 8.3 GHz for *eV*_B_/(2Δ) > 1 and becomes taller with increasing bias voltage. Figure 4(b) exhibits the traces of the spectral density, which are integrated for Fig. 4(c) to obtain the generated power as a function of the bias voltage. The qualitative behavior of the generated power matches that of Sample A: the power begins to increase with bias voltage around *eV*_B_/(2Δ) = 1, where we observe a shallow dip corresponding to cooling of the resonator. However, the magnitude of the generated power is greater and the mode temperature shown in Fig. 4(d) is lower compared with Fig. 2. This is because the coupling of the resonator to the transmission line is proportional to the third power of the resonance frequency [see Eq. (1)] which is almost twice as large here in comparison to Sample A.

## Conclusions

We observed microwave absorption and emission arising from photon-assisted single-electron tunneling through NIS junctions. We experimentally verified cooling by the quantum-circuit refrigerator^14^ by measuring for the first time the power spectral density of microwaves emitted from the resonator. Furthermore, we have implemented an incoherent microwave source which realizes direct conversion of the electrostatic energy provided by the dc voltage source to microwave photons. Importantly, it does not rely on the thermal voltage fluctuation spectrum of a resistor, and much like shot-noise sources^19,39^, it may provide higher effective temperatures, faster tunability, and less excess heating than usual thermal methods. Our measurements of the generated power are in good agreement with the corresponding theoretical model, heavily suggesting that our interpretation that photon-assisted tunneling is mainly responsible for the generated power is correct. This interpretation is verified by the observation of much higher generated powers than what is expected for resonator temperatures matching the normal-metal electron temperature. In the future, we aim to extend this photon source to narrow bandwidths and pulsed operation. In an effort to obtain a stringent quantitative verification of the validity of the used theoretical model, we aim to measure the characteristics of the source using a cryogenic microwave bolometer^10^ or a qubit coupled to the resonator^52^.

## Electronic supplementary material

Supplementary information
