# Supplementary material for: Observation of microwave absorption and emission from incoherent electron tunneling through a normal-metal–insulator–superconductor junction
Source: Sci Rep. 2018 Mar 2;8:3966. doi: 10.1038/s41598-018-21772-5 (PMC5834461; doi:10.1038/s41598-018-21772-5)
Supplement: Supplementary file 1 — Supplementary information [file 41598_2018_21772_MOESM1_ESM.pdf]

Supporting information: Observation of microwave absorption and emission from incoherent electron tunneling through a normal-metal–insulator–superconductor junction

Shumpei Masuda[1,*], Kuan Y. Tan[1], Matti Partanen[1], Russell E. Lake[1], Joonas Govenius[1], Matti Silveri[1,2], Hermann Grabert[3] and Mikko Möttönen[1]

[1] QCD Labs, COMP Centre of Excellence, Department of Applied Physics, Aalto University, PO Box 13500, FI-00076 Aalto, Finland

[2] Research Unit of Theoretical Physics, University of Oulu, FI-90014 Oulu, Finland

[3] Department of Physics, University of Freiburg, Germany

E-mail: masulas@tmd.ac.jp; mikko.mottonen@aalto.fi

I. PARAMETERS

The fundamental resonance frequency of a homogeneous half-wavelength CPW resonator of length $L_{\rm res}$ is given by

$$f_0 = \frac{c}{2L_{\rm res}\sqrt{\varepsilon_{\rm eff}}} \tag{S1}$$

where the speed of light in vacuum is denoted by c and the effective relative permittivity of the resonator by $\varepsilon_{\rm eff}$. The CPW capacitance $c_{\rm res}$ and inductance $l_{\rm res}$ per unit length can be expressed as [53, 54]

$$\begin{aligned} c_{\rm res} &= 4\varepsilon_0\varepsilon_{\rm eff}\frac{K(k_0)}{K(k_0')}, \\ l_{\rm res} &= \frac{\mu_0 K(k_0')}{4K(k_0)}, \end{aligned} \tag{S2}$$

where K is the complete elliptic integral of the first kind and

$$\begin{aligned} k_0 &= \frac{w}{w+2s}, \\ k_0' &= \sqrt{1-k_0^2}, \end{aligned} \tag{S3}$$

depend on the width of the center conductor w, and on the separation between the center conductor and the ground plane of the CPW resonator s. We calculate c_{res} and l_{res} in Eq. (S2) using Eqs. (S1) and (S3) and the measured values for f_0, L_{res}, w, and s given in Table I. We neglect the kinetic inductance because it is sufficiently smaller than the geometric inductance in Eq. (S2) [54], although in principle, the total inductance per unit length is the sum of the geometric and the kinetic contributions. The resonance frequency calculated using Eq. (S1) and the analytic form of $\varepsilon_{\mathrm{eff}}$ for an unshielded CPW [53] has less than 1% difference from the measured value for Sample A and about 4% deviation for Sample B. The characteristic impedance Z_0 of the transmission line is obtained from $Z_0 = \sqrt{l_{\mathrm{res}}/c_{\mathrm{res}}}$.

Capacitances C_k for $k \in \{1, 2, 3, 4\}$ are estimated using

$$C_k = \frac{A_k \varepsilon}{d}, \tag{S4}$$

where A_k is the area of the parallel-plate capacitor, the permittivity of the aluminum oxide dielectric is denoted by $\varepsilon \approx 9.8 \times \varepsilon_0$, where ε_0 is the permittivity of vacuum, and $d = 50$ nm is the thickness of the aluminum oxide layer. The capacitance density for C_{J} is 45 fF/μm^2. The tunnel junction area is approximately 200×200 nm^2 inferred from the SEM image of the sample.

In the absence of an electromagnetic environment, the current through an NIS junction is given by

$$\begin{aligned} I(V_{\mathrm{B}}) = \frac{1}{eR_{\mathrm{T}}} \int_{-\infty}^{\infty} \mathrm{d}E \Big[& n_{\mathrm{S}}(E) f(E, T_{\mathrm{S}}) [1 - f(E + eV_{\mathrm{B}}/2, T_{\mathrm{N}})] \\ & - n_{\mathrm{S}}(E - eV_{\mathrm{B}}/2)[1 - f(E - eV_{\mathrm{B}}/2, T_{\mathrm{S}})] f(E, T_{\mathrm{N}}) \Big], \end{aligned} \tag{S5}$$

where T_{N} is the electron temperature of the normal-metal island obtained using the thermometer junctions and f is the Fermi–Dirac distribution function

$$f(E, T) = \frac{1}{e^{E/(k_{\mathrm{B}}T)} + 1}. \tag{S6}$$

The superconductor density of states n_{S} is represented by

$$n_{\mathrm{S}}(E) = \left| \mathrm{Re} \Big[\frac{E/\Delta + i\gamma_{\mathrm{D}}}{\sqrt{(E/\Delta + i\gamma_{\mathrm{D}})^2 - 1}} \Big] \right|, \tag{S7}$$

where γ_D is the Dynes parameter [15]. The tunnel resistance R_T, the Dynes parameter, and the superconductor gap parameter Δ are obtained from a fit of Eq. (S5) to measured junction IV characteristics.

II. APPARENT TRANSMISSION LINE TEMPERATURE

In our thermal model in Fig. 3, the resonator is thermally connected only to the transmission line and to the tunneling electrons. Due to the simple way the transmission line heats the resonator given by Eq. (1) however, any additional constant heating power to the resonator is accounted in this model by an adjustment of the average photon number of the transmission line. This gives rise to the apparent temperature of the transmission line, T_{TL}, connected to the average photon number, $\bar{n}_{TL}$, through the Bose distribution function. Note that $\bar{n}_{TL}$ is independent of the bias voltage since also the electron temperature of the 50-Ω resistor in Fig. 1(d) giving rise to the photons incident on the resonator does not change with the bias voltage.

Since the power arising from the photon-assisted tunneling is negligible at zero bias voltage, we have $P_{RT} = 0$ in Eq. (1), and hence $T_{res} = T_{TL}$ at zero bias. Thus if we find T_{TL} at any bias voltage, we obtain the temperature and the average photon number of the resonator at zero bias. Consequently, we obtain these quantities at every bias voltage point using Eq. (2) and the directly measured power difference between the finite and zero bias points.

We use the bias point, at which the measured generated power crosses zero, as the calibration voltage for T_{TL}. Here, the net exchanged power given by Eq. (1) and the temperature of the resonator coincides with that at zero bias. Thus, the power arising from photon-assisted tunneling, P_{JR}, must vanish. As we show in the next section, this point is independent of many of the sample parameters such as the tunnel resistance, R_T. It is also independent of the gain of our amplification chain. Thus, our model accurately yields the resonator temperature at the calibration point which equals the apparent transmission line temperature. We attribute the elevated T_{TL} in Table I to heating of the 50-Ω termination in Fig. 1(d) owing to the thermal and amplifier noise and to possible leakage radiation to the sample holder.

III. ENERGY TRANSFER BETWEEN TUNNELING ELECTRONS AND THE RESONATOR

The electron tunneling may lead to creation and annihilation of photons at the resonator giving rise to the power transfer P_{JR} in Fig. 3. To this end, we employ the $P(E)$ theory which has been developed to obtain the electron tunneling rates across tunnel junctions and the resulting energy transfer to the environment [30]. In terms of the $P(E)$ theory, the environment refers to the electrical degrees of freedom which are coupled to the tunneling process.

The power transfer to the environment strongly depends on the electrical impedance of the system. A simplified electrical circuit diagram of our system is depicted in Fig. S1(a). The first harmonic mode of the CPW resonator is modeled as a parallel LC circuit with capacitance $C_{\mathrm{L}} = c_{\mathrm{res}} L_{\mathrm{res}}/2$, inductance $L_{\mathrm{L}} = 2 l_{\mathrm{res}} L_{\mathrm{res}}/\pi^2$ and angular frequency $\omega_0 = 1/\sqrt{C_{\mathrm{L}} L_{\mathrm{L}}}$. We assume that the electron tunneling event on either of the junctions is hardly affected by another junction because of a small junction capacitance C_{J} and large tunnel resistance R_{T}, and that the direct influence of the transmission line to the electron tunneling is neglected due to a relatively small coupling capacitance C_4. Thus, we consider a pair of separate effective circuits depicted in Figs. S1(b) and S1(c) to calculate the tunneling rate at each NIS junction. We assume that the resistance of the normal-metal island is small enough to be neglected because it is in series with the small junction capacitance, and hence negligible current flows through it with respect to Ohmic losses.

The power from the tunneling electrons to the environment is represented by

$$P_{\mathrm{env}}(V_{\mathrm{B}}/2, T_{\mathrm{S}}, T_{\mathrm{N}}) = 2[\overrightarrow{P}(V_{\mathrm{B}}/2, T_{\mathrm{S}}, T_{\mathrm{N}}) + \overleftarrow{P}(V_{\mathrm{B}}/2, T_{\mathrm{S}}, T_{\mathrm{N}})], \tag{S8}$$

where the powers caused by the forward and the backward electron tunneling events at each junction are given by [30]

$$\begin{aligned}\overrightarrow{P}(V_{\mathrm{B}}/2, T_{\mathrm{S}}, T_{\mathrm{N}}) &= \frac{1}{e^2 R_{\mathrm{T}}} \iint_{-\infty}^{\infty} \mathrm{d}E \mathrm{d}E' n_{\mathrm{S}}(E) f(E, T_{\mathrm{S}})[1 - f(E' + eV_{\mathrm{B}}/2, T_{\mathrm{N}})] \\ &\quad \times (E - E') P(E - E', T_{\mathrm{env}}), \\ \overleftarrow{P}(V_{\mathrm{B}}/2, T_{\mathrm{S}}, T_{\mathrm{N}}) &= \frac{1}{e^2 R_{\mathrm{T}}} \iint_{-\infty}^{\infty} \mathrm{d}E \mathrm{d}E' n_{\mathrm{S}}(E' - eV_{\mathrm{B}}/2)[1 - f(E' - eV_{\mathrm{B}}/2, T_{\mathrm{S}})] f(E, T_{\mathrm{N}}) \\ &\quad \times (E - E') P(E - E', T_{\mathrm{env}}),\end{aligned} \tag{S9}$$

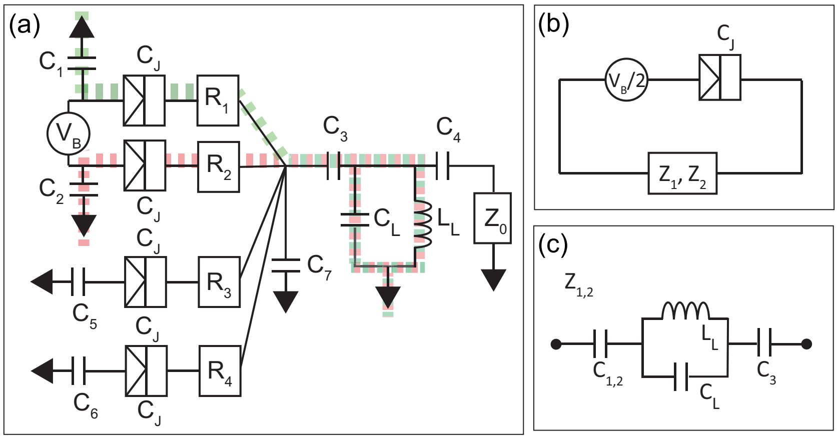

FIG. S1. (a) Circuit model of the realized microwave source. The resonator is modelled by a single parallel LC circuit. See Fig. 1 for the definition of the capacitances. The resistances arise from the finite conductance of the normal-metal island of the NIS junctions. (b) Effective circuits for photon-assisted tunneling at each NIS junction. The systems with Z_1 and Z_2 correspond to the red and green conduction paths in panel (a), respectively. (c) Composition of the lumped elements forming Z_1 and Z_2. Here, the finite conductance of the normal-metal island and the direct interplay between the electron tunneling and losses to the transmission line described by Z_0 have been neglected.

respectively. Here, T_S and T_env denote the temperatures of the superconductor and of the environment, respectively. The factor of two in Eq. (S8) arises from the fact that the two effective circuits in Fig. S1(b) have an identical contribution to the power. The probability density $P(E)$ for the environment to absorb energy E during a tunneling event is expressed as [30]

$$P(E, T_\mathrm{env}) = \frac{1}{2\pi\hbar}\int_{-\infty}^{\infty} \mathrm{d}t \exp[J(t, T_\mathrm{env})] \exp\left[i\frac{E}{\hbar}t\right], \tag{S10}$$

where

$$J(t, T_\mathrm{env}) = 2\int_0^{\infty} \frac{\mathrm{d}\omega}{\omega}\frac{\mathrm{Re}[Z_\mathrm{t}(\omega)]}{R_\mathrm{K}}\left[\coth\left(\frac{\hbar\omega}{2k_\mathrm{B}T_\mathrm{env}}\right)[\cos(\omega t) - 1] - i\sin(\omega t)\right], \tag{S11}$$

and

$$\mathrm{Re}[Z_\mathrm{t}] = \frac{\pi}{2C_\mathrm{L}}\left[\delta(\omega - \omega_0) + \delta(\omega + \omega_0)\right] + \frac{\pi}{2}\frac{C_{1(2)} + C_3}{C_{1(2)}C_3}\delta(\omega). \tag{S12}$$

In $P(E)$ theory, the effective impedance Z_t in Eq. (S11) is the impedance of the external circuit $Z_{1/2}$ in parallel with C_J. The influence of C_J, which is approximately 3 fF $\ll C_{1/2/3}$,

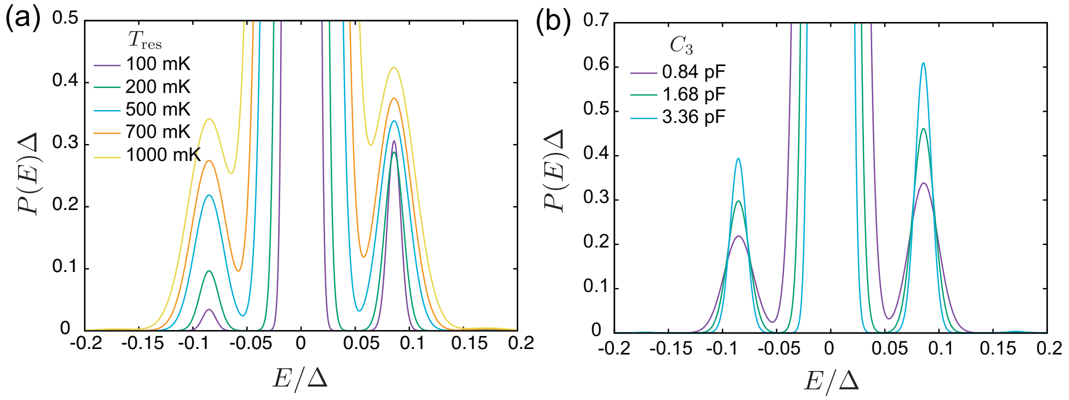

FIG. S2. (a) $P(E)$ function of Sample A for $eV_{\rm B}/(2\Delta) = 1.2$ and $T_{\rm res}$ as indicated. The other parameters are given in Table I. (b) $P(E)$ of Sample A shown for various indicated C_3, for $T_{\rm res} = 500$ mK and $eV/(2\Delta) = 1.2$.

to the effective impedance is neglected in Eq. (S12) because $Z_{\rm t}$ is dominated by $Z_{1/2}$. The influence of $C_{1/2}$ is also negligible because it is more than 50 times larger than C_3 and approximately cancel out in Eq. (S12).

Note that the environment consists not only of the resonator but also of the capacitors $C_{1/2/3}$. Finite $C_{1/2/3}$ broadens the peaks of $P(E)$ which become delta functions in the limit of infinite $C_{1/2/3}$. Figure S2(a) shows $P(E)$ of Sample A for various $T_{\rm res}$ at $eV_{\rm B}/(2\Delta) = 1.2$, where we assumed $T_{\rm env} = T_{\rm res}$. The peaks at $E = \hbar\omega_0 \approx 0.09\times\Delta$ and $E = -\hbar\omega_0 \approx -0.09\times\Delta$ correspond to the emission and absorption of a single photon from the resonator, respectively. The high peak at $E = 0$ corresponds to the elastic tunnelling. We assume that only the peaks at $|E| = \pm\hbar\omega_0$ contribute to $P_{\rm JR}$. Thus, only a part of $P_{\rm env}$ contributes to $P_{\rm JR}$. We compute $P_{\rm JR}$ using Eqs. (S8) and (S9) where we first replace P by

$$P_{\rm M}(E, T_{\rm res}) = \begin{cases} P(E, T_{\rm res}) & \text{for } |E| \geq \hbar\omega_0/2, \\ 0 & \text{for} |E| < \hbar\omega_0/2. \end{cases} \tag{S13}$$

As shown in Fig. S2(a), the peaks become broader with increasing $T_{\rm res}$. The center peak and the side peaks are not well separated for high $T_{\rm res}$. Thus, the numerical results of Sample A may have considerable error beyond $eV/(2\Delta) = 1.4$ corresponding to $T_{\rm res} >$ 500 mK [see Fig. 2(d)]. The minor discrepancy between the numerical and experimental results in Fig. 2(c) is attributed to this fact. Figure S2(b) shows the dependence of $P(E)$ on the coupling capacitance C_3. The peak becomes narrower for larger C_3 due to the increasing

coupling strength between a tunneling electron and the resonator.

IV. ENERGY TRANSFER BETWEEN THE RESONATOR AND THE TRANSMISSION LINE

Let us study a model for the energy transfer between a CPW resonator and a transmission line which are mutually capacitively coupled as shown in Fig. S3. First, we model the transmission line as a CPW resonator of finite length and finally extend the length to infinity. The electrostatic energy of the coupling capacitor C_4 is given by the voltage difference between the resonator and the transmission line as $C_4(V_{\rm TL} - V_{\rm res})^2/2$, where $V_{\rm res}$ and $V_{\rm TL}$ are the voltage of the resonator and the transmission line at the capacitor, respectively. Thus, the interaction Hamiltonian between the resonator and the transmission line is represented as

$$\hat{H}_{\rm int} = -C_4 \hat{V}_{\rm res} \hat{V}_{\rm TL}. \tag{S14}$$

The voltage operators of the resonator $\hat{V}_{\rm res}$ and of the transmission line $\hat{V}_{\rm TL}$ are represented as $\hat{V}_{\rm res} = \sum_M V_M^{(\rm res)}(\hat{a}_M^\dagger + \hat{a}_M)$ and $\hat{V}_{\rm TL} = \sum_N V_N^{(\rm TL)}(\hat{b}_N^\dagger + \hat{b}_N)$, respectively, with the creation operator $\hat{a}_M^\dagger$ and the annihilation operator $\hat{a}_M$ of a photon in the Mth mode of the resonator and the creation operator $\hat{b}_N^\dagger$ and the annihilation operator $\hat{b}_N$ of a photon in the Nth mode of the transmission line [2]. The coefficients are written as $V_M^{(\rm res)} = \sqrt{M\hbar\omega_{\rm res}/(L_{\rm res}c_{\rm res})}$ and $V_N^{(\rm TL)} = \sqrt{N\hbar\omega_{\rm TL}/(L_{\rm TL}c_{\rm TL})}$ with the fundamental resonance frequencies $\omega_{\rm res/TL} = \pi/(L_{\rm res/TL}\sqrt{l_{\rm res/TL}c_{\rm res/TL}})$. The length of the resonator (transmission line) is $L_{\rm res}$ ($L_{\rm TL}$), the capacitance per unit length is $c_{\rm res}$ ($c_{\rm TL}$) and the inductance per unit length is $l_{\rm res}$ ($l_{\rm TL}$). Above, we have assumed that C_4 is so small that its effect on $\omega_{\rm res/TL}$ and the operators may be neglected.

We employ a basis composed of the energy eigenstates of an uncoupled resonator–transmission-line system. Assuming weak coupling, we may estimate the transition rate from an energy eigenstate $|i\rangle = |m_1, m_2, \cdots ; n_1, n_2, \cdots\rangle$ to a different one $|f\rangle = |m_1', m_2', \cdots ; n_1', n_2', \cdots\rangle$ using Fermi's golden rule, where m_M (n_N) denotes the number of photons in the Mth (Nth)

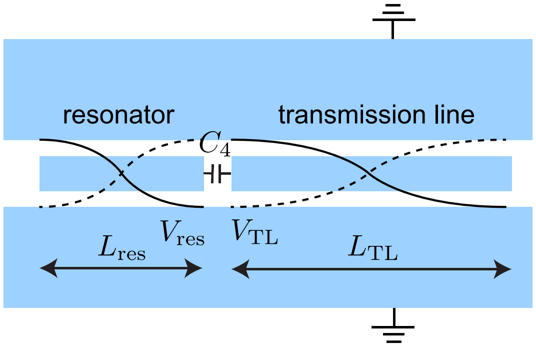

FIG. S3. Illustration of a CPW resonator and a transmission line coupled by capacitance C_4. The solid and the dashed lines represent the voltage difference between the center conductor and the ground plane. The transmission line is modelled by a CPW resonator with length $L_{\rm TL}$ which is eventually extended to infinity.

mode of the resonator (transmission line). The Hamiltonian in Eq. (S14) is rewritten as

$$\hat{H}_{\rm int}^{\rm eff} = -C_4 \sum_M \sum_N V_M^{(\rm res)} V_N^{(\rm TL)} (\hat{a}_M^\dagger \hat{b}_N + \hat{a}_M \hat{b}_N^\dagger), \tag{S15}$$

where we omitted the terms proportional to $\hat{a}_M^\dagger \hat{b}_N^\dagger$ and $\hat{a}_M \hat{b}_N$ which do not contribute to the transition rates in Fermi's golden rule. We first consider the case where a photon in the Ith mode of the resonator is annihilated and a photon in the Jth mode of the transmission line is created, i.e., we only consider the term $-C_4 V_I^{(\rm res)} V_J^{(\rm TL)} \hat{a}_I \hat{b}_J^\dagger$ in Eq. (S15). Consequently, the probability $P_{if}(t)$ that we find the state $|f\rangle$ at time t starting from $|i\rangle$ is given by Fermi's golden rule as

$$\begin{aligned} P_{if}^{(IJ)}(t) = {} & t\frac{2\pi}{\hbar} C_4^2 (V_I^{(\rm res)})^2 (V_J^{(\rm TL)})^2 m_I (n_J+1) \\ & \times \delta(E_f - E_i) \delta_{m_I', m_I - 1} \delta_{n_J', n_J+1} \Big(\prod_{K \neq I} \delta_{m_K', m_K} \Big) \Big(\prod_{K \neq J} \delta_{n_K', n_K} \Big), \end{aligned} \tag{S16}$$

where $E_f - E_i = \hbar(\omega_{\rm TL}^{(J)} - \omega_{\rm res}^{(I)})$ with $\omega_{\rm TL}^{(J)} = J\omega_{\rm TL}$ and $\omega_{\rm res}^{(I)} = I\omega_{\rm res}$.

Let us calculate the probability $P_I^{(+)}(t)$ that a photon in the Ith mode of the resonator is annihilated and a photon in the transmission line is created. To this end, we sum the probabilities for all photon numbers and every mode J in the transmission line. First, we execute the summation over the final photon numbers, which results in

$$P_i^{(IJ)} = t\frac{2\pi}{\hbar} C_4^2 (V_I^{(\rm res)})^2 (V_J^{(\rm TL)})^2 m_I (n_J+1) \delta[\hbar(\omega_{\rm TL}^{(J)} - \omega_{\rm res}^{(I)})]. \tag{S17}$$

Next we sum with respect to J in the limit where L_{TL} extends to infinity. Thus, we replace the discrete summation over J by an integral with respect to energy using the relation

$$\lim_{L_{\mathrm{TL}}\to\infty} \frac{1}{\Delta E_J} \sum_J \Delta E_J = \lim_{L_{\mathrm{TL}}\to\infty} \frac{1}{\Delta E_J} \int_0^\infty \mathrm{d}E_J, \tag{S18}$$

where $\Delta E_J = \hbar\omega_{\mathrm{TL}} = \hbar\pi/(L_{\mathrm{TL}}\sqrt{l_{\mathrm{TL}}c_{\mathrm{TL}}})$. Using Eq. (S18) to sum over J in Eq. (S17), the probability of a photon in the Ith mode to decay given the initial state $|i\rangle$ is represented by

$$\begin{aligned} P_i^{(I)}(t) &= \lim_{L_{\mathrm{TL}}\to\infty} \sum_J P_i^{(IJ)}(t) \\ &= \frac{2L_{\mathrm{TL}}\sqrt{l_{\mathrm{TL}}c_{\mathrm{TL}}}}{\hbar^2} C_4^2 (V_I^{(\mathrm{res})})^2 (V_J^{(\mathrm{TL})})^2 m_\omega (n_\omega + 1)t \\ &= 2C_4^2 \frac{\omega^2 Z_0}{L_{\mathrm{res}}c_{\mathrm{res}}} m_\omega (n_\omega + 1)t, \end{aligned} \tag{S19}$$

where $Z_0 = \sqrt{l_{\mathrm{TL}}/c_{\mathrm{TL}}}$ and we have introduced a notation $m_\omega := m_I$ and n_ω is the photon number in the initial state of the transmission line corresponding to the mode with angular frequency $\omega := \omega_{\mathrm{res}}^{(I)}$.

Considering all the possible initial states with different photon numbers, the probability of annihilating a photon in the resonator mode I, $P_I^{(+)}$, may be represented as

$$\begin{aligned} P_\omega^{(+)}(t) := P_I^{(+)}(t) &= \sum_{m_\omega} \sum_{n_\omega} p_{m_\omega} p_{n_\omega} P_i^{(I)}(t) \\ &= 2C_4^2 \frac{\omega^2 Z_0}{L_{\mathrm{res}}c_{\mathrm{res}}} \bar{m}_\omega (\bar{n}_\omega + 1)t, \end{aligned} \tag{S20}$$

where the average photon numbers with angular frequency ω in the resonator and the transmission line are denoted by $\bar{m}_\omega$ and $\bar{n}_\omega$, respectively. Here, p_{m_ω} (p_{n_ω}) is the probability that there are m_ω (n_ω) photons with angular frequency ω in the resonator (transmission line) in the initial state. Hereafter, we replace $P_I^{(+)}$ by $P_\omega^{(+)}$ for simplicity of notation. Thus, the rate that photons in the resonator with the energy $\hbar\omega$ transfer to the transmission line is obtained as

$$\Gamma_{\mathrm{res,TL}}^{(\omega)} = \frac{\mathrm{d}P_\omega^{(+)}}{\mathrm{d}t} = 2C_4^2 \frac{\omega^2 Z_0}{L_{\mathrm{res}}c_{\mathrm{res}}} \bar{m}_\omega (\bar{n}_\omega + 1). \tag{S21}$$

In the same manner the photon transition rate from the transmission line to the resonator

is obtained as

$$\Gamma_{\mathrm{TL,res}}^{(\omega)} = 2C_4^2 \frac{\omega^2 Z_0}{L_{\mathrm{res}} c_{\mathrm{res}}} (\bar{m}_\omega + 1)\bar{n}_\omega. \tag{S22}$$

The net power flow from the resonator to the transmission line mediated by the photons with angular frequency ω is represented with the photon numbers in the resonator and the transmission line as

$$\begin{aligned} P_{\mathrm{RT}}^{(\omega)} = \hbar\omega[\Gamma_{\mathrm{res,TL}} - \Gamma_{\mathrm{TL,res}}] &= 2C_4^2 \frac{\hbar\omega^3 Z_0}{L_{\mathrm{res}} c_{\mathrm{res}}} (\bar{m}_\omega - \bar{n}_\omega) \\ &= C_4^2 \frac{\hbar\omega^3 Z_0}{C_{\mathrm{L}}} (\bar{m}_\omega - \bar{n}_\omega). \end{aligned} \tag{S23}$$

In the thermal state, we have

$$\begin{aligned} \bar{m}_\omega &= \frac{1}{e^{\hbar\omega/(k_{\mathrm{B}} T_{\mathrm{res}})} - 1}, \\ \bar{n}_\omega &= \frac{1}{e^{\hbar\omega/(k_{\mathrm{B}} T_{\mathrm{TL}})} - 1}, \end{aligned} \tag{S24}$$

and hence

$$P_{\mathrm{RT}}^{(\omega)} = C_4^2 \frac{\hbar\omega^3 Z_0}{C_{\mathrm{L}}} \Big[\frac{1}{e^{\hbar\omega/(k_{\mathrm{B}} T_{\mathrm{res}})} - 1} - \frac{1}{e^{\hbar\omega/(k_{\mathrm{B}} T_{\mathrm{TL}})} - 1} \Big], \tag{S25}$$

owing to Eq. (S23).

V. THERMOMETRY

A pair of current-biased NIS junctions is used as a thermometer as illustrated in Fig. 1(b). The voltage V_{th} across the thermometer junctions provides a good measure of the electron temperature T_{N} of the normal-metal island in the temperature range of interest to us. In $P(E)$ theory, the tunnel current is written as [30]

$$\begin{aligned} I_{\mathrm{th}}(V_{\mathrm{th}}, T_{\mathrm{N}}, T_{\mathrm{S}}, T_{\mathrm{env}}) = \frac{1}{e R_{\mathrm{T}}} \iint_{-\infty}^{\infty} \mathrm{d}E \mathrm{d}E' \Big[& n_{\mathrm{S}}(E) f(E, T_{\mathrm{S}}) [1 - f(E' + eV_{\mathrm{th}}/2, T_{\mathrm{N}})] \\ & - n_{\mathrm{S}}(E' - eV_{\mathrm{th}}/2)[1 - f(E' - eV_{\mathrm{th}}/2, T_{\mathrm{S}})] f(E, T_{\mathrm{N}}) \Big] P(E - E', T_{\mathrm{env}}). \end{aligned} \tag{S26}$$

The current in Eq. (S26) is approximated by

$$I_{\rm th}(V_{\rm th},T_{\rm N}) = \frac{1}{eR_{\rm T}}\int_{-\infty}^{\infty} {\rm d}E\Big[n_{\rm S}(E)f(E,T_{\rm S})[1-f(E+eV_{\rm th}/2,T_{\rm N})] \\ -n_{\rm S}(E-eV_{\rm th}/2)[1-f(E-eV_{\rm th}/2,T_{\rm S})]f(E,T_{\rm N})\Big], \qquad \text{(S27)}$$

where we replaced $P(E-E',T_{\rm env})$ by $\delta(E-E')$ because in our case, the current is dominated by the elastic electron tunneling. Here, we neglect the dependence of $I_{\rm th}$ on $T_{\rm S}$ justified by $k_{\rm B}T_{\rm S} \ll \Delta$. Thus, the voltage $V_{\rm th}$ essentially depends only on $I_{\rm th}$ and $T_{\rm N}$. For a fixed value of $I_{\rm th}$, $V_{\rm th}$ can be regarded as a single-valued function of $T_{\rm N}$. We utilize this property to convert the measured $V_{\rm th}$ into $T_{\rm N}$.

Figure S4(a) shows the thermometer voltage as a function of the bath temperature T_0 measured for $I_{\rm th}$ =17.7 pA and vanishing bias voltage at the other junctions, $V_{\rm B}=0$. The experimental value of $V_{\rm th}(T_0)$ matches the theoretical value obtained from Eq. (S27) for $V_{\rm th}(T_{\rm N}=T_0)$ and $T_0 > 100$ mK. This is because the electron temperature of the normal-metal island is close to the bath temperature for high T_0. The agreement of the theoretical and numerical results shows that this model of the thermometer is accurate. On the other hand, the experimental values of $V_{\rm th}$ are lower than the theoretical ones at $T_0 < 100$ mK since the electrons thermally decouple from the phonons leading to a saturation of the electron temperature [15]. Neverthless, the theoretical model can be used to convert the measured $V_{\rm th}$ to $T_{\rm N}$ even in this temperature range and under finite $V_{\rm B}$. Figure S4(b) shows the measured $T_{\rm N}$ for several bias voltages obtained using the calibration data in Fig. S4(a).

---

[1] Wolf, E. L. *Quantum Nanoelectronics: An Introduction to Electronic Nanotechnology and Quantum Computing* (Wiley-VCH, Weinheim, 2009).

[2] Blais, A., Huang, R.-S., Wallraff, A., Girvin, S. M. & Schoelkopf, R. J. Cavity Quantum Electrodynamics for Superconducting Electrical Circuits: an Architecture for Quantum Computation. *Phys. Rev. A* **69,** 062320 (2004).

[3] Wallraff, A., Schuster, D. I., Blais, A., Frunzio, L., Huang, R.-S., Majer, J., Kumar, S., Girvin, S. M. & Schoelkopf, R. J. Strong Coupling of a Single Photon to a Superconducting Qubit using Circuit Quantum Electrodynamics. *Nature* **431,** 162–167 (2004).

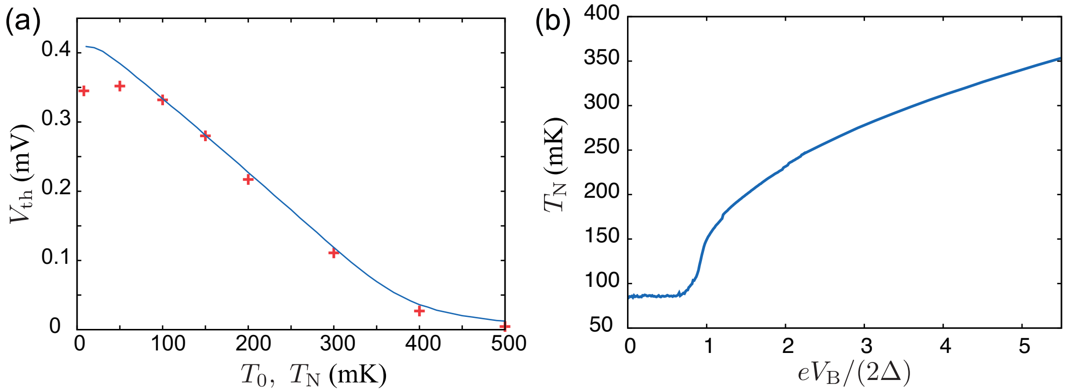

FIG. S4. (a) Measured voltage (markers) $V_{\rm th}$ across the two thermometer junctions of Sample A as a function of the phonon bath temperature T_0 for $V_{\rm B} = 0$ and $I_{\rm th}$ =17.7 pA. The solid line shows $V_{\rm th}$ as a function of the electron temperature $T_{\rm N}$ as calculated using Eq. (S27). This curve serves as the conversion function from $V_{\rm th}$ into $T_{\rm N}$. (b) Measured electron temperature as a function of the bias voltage $V_{\rm B}$ in the case of Figs. 2(c) and 2(d).

[4] Majer, J., Chow, J. M., Gambetta, J. M., Koch, J., Johnson, B. R., Schreier, J. A., Frunzio, L., Schuster, D. I., Houck, A. A., Wallraff, A., Blais, A., Devoret, M. H., Girvin, S. M. & Schoelkopf, R. J. Coupling Superconducting Qubits via a Cavity Bus. *Nature* **449,** 443–447 (2007).

[5] Sillanpää, M. A., Park, J. I & Simmonds, R. W. Coherent Quantum State Storage and Transfer between Two Phase Qubits via a Resonant Cavity. *Nature* **449,** 438–442 (2007).

[6] Devoret, M. H. & Schoelkopf, R. J. Superconducting Circuits for Quantum Information: an Outlook. *Science* **339,** 1169–1174 (2013).

[7] Kelly, J. *et al.* State Preservation by Repetitive Error Detection in a Superconducting Quantum Circuit. *Nature* **519,** 66–69 (2015).

[8] Ofek, N. *et al.* Extending the Lifetime of a Quantum Bit with Error Correction in Superconducting Circuits. *Nature* **536,** 441–445 (2016).

[9] Inomata, K., Lin, Z. R., Koshino, K., Oliver, W. D., Tsai, J. S., Yamamoto, T. & Nakamura, Y. Single Microwave-Photon Detector using an Artificial Λ-Type Three-Level System. *Nat. Commun.* **7,** 12303 (2016).

[10] Govenius, J., Lake, R. E., Tan, K. Y. & Mttnen, M. Detection of Zeptojoule Microwave Pulses using Electrothermal Feedback in Proximity-Induced Josephson Junctions. *Phys. Rev. Lett.* **117,** 030802 (2016).

[11] Saira, O.-P., Zgirski, M., Viisanen, K. L., Golubev, D. S. & Pekola, J. P. Dispersive Thermometry with a Josephson Junction Coupled to a Resonator. *Phys. Rev. Applied* **6,** 024005 (2016).

[12] Clark, A. M., Miller, N. A., Williams, S., Ruggiero, S. T., Hilton, G. C., Vale, L. R., Beall, K. D., Irwin, K. D. & Ullom, J. N. Cooling of Bulk Material by Electron-Tunneling Refrigerators. *Appl. Phys. Lett.* **86,** 173508–173510 (2005).

[13] Timofeev, A. V., Helle, M., Meschke, M., Möttönen, M. & Pekola, J. P. Electronic Refrigeration at the Quantum Limit. *Phys. Rev. Lett.* **102,** 200801 (2009).

[14] Tan, K. Y., Partanen, M., Lake, R. E., Govenius, J., Masuda, S. & Möttönen, M. Quantum-circuit Refrigerator. *Nat. Commun.* **8,** 15189 (2017).

[15] Giazotto, F., Heikkilä, T. T., Luukanen, A., Savin, A. M. & Pekola, J. P. Opportunities for Mesoscopics in Thermometry and Refrigeration: Physics and Applications. *Rev. Mod. Phys.* **78,** 217–274 (2006).

[16] Hofheinz, M., Wang, H., Ansmann, M., Bialczak, R. C., Lucero, E., Neeley, M., O'Connell, A. D., Sank, D., Wenner, J., Martinis, J. M. & Cleland, A. N. Synthesizing Arbitrary Quantum States in a Superconducting Resonator. *Nature* **459,** 546–549 (2009).

[17] Meschke, M., Guichard, W. & Pekola, J. P. Single-mode Heat Conduction by Photons. *Nature* **444,** 187–190 (2006).

[18] Partanen, M., Tan, K. Y., Govenius, J., Lake, R. E., Mäkelä, K., Tanttu, T. & Möttönen, M. Quantum-Limited Heat Conduction over Macroscopic Distances. *Nat. Phys.* **12,** 460–464 (2016).

[19] Spietz, L. *et al.* Primary Electronic Thermometry Using the Shot Noise of a Tunnel Junction. *Science* **300,** 1929–1932 (2003).

[20] Goetz, J., Pogorzalek, S., Deppe, F., Fedorov, K. G., Eder, P., Fischer, M., Wulschner, F., Xie, E., Marx, A. & Gross, R. Photon Statistics of Propagating Thermal Microwaves. *Phys. Rev. Lett.* **118,** 103602 (2016).

[21] Spietz, L. *et al.* Noise performance of lumped element direct current superconducting quantum interference device amplifiers in the 4–8 GHz range. *Applied Phys. Lett.* **97,** 142502 (2010).

[22] Chen, F. *et al.* Realization of a single-Cooper-pair Josephson laser. *Phys. Rev. B* **90,** 020506 (2014).

[23] Cassidy, M. C. *et al.* Demonstration of an ac Josephson junction laser. *Science* **355,** 939–942

(2017).

[24] Houck, A. A., Schuster, D. I., Gambetta, J. M., Schreier, J. A., Johnson, B. R., Chow, J. M., Frunzio, L., Majer, J., Devoret, M. H., Girvin, S. M. & Schoelkopf, R. J. Generating Single Microwave Photons in a Circuit. *Nature* **449,** 328–331 (2007).

[25] Bozyigit, D. *et al.* Antibunching of microwave-frequency photons observed in correlation measurements using linear detectors. *Nat. Phys.* **7** 154–158 (2011).

[26] Peng, Z. H., de Graaf, S. E., Tsai, J. S. & Astafiev, O. V. *Nat. Commun.* Tuneable on-demand single-photon source in the microwave range. **449,** 12588 (2016).

[27] Devoret, M. H., Esteve, D., Grabert, H., Ingold, G.-L., Pothier, H. & Urbina, C. Effect of the Electromagnetic Environment on the Coulomb Blockade in Ultrasmall Tunnel Junctions. *Phys. Rev. Lett.* **64,** 1824 (1990).

[28] Girvin, S. M., Glazman, L. I., Jonson, M., Penn, D. R. & Stiles, M. D. Quantum Fluctuations and the Single-Junction Coulomb Blockade. *Phys. Rev. Lett.* **64,** 3183 (1990).

[29] Averin, B., Nazarov, Y. & Odintsov, A. Incoherent Tunneling of the Cooper Pairs and Magnetic Flux Quanta in Ultrasmall Josephson Junctions. *Physica B* **165/166,** 945–946 (1990).

[30] Ingold, G.-L., & Nazarov, Y. V. Charge Tunneling Rates in Ultrasmall Junctions. *NATO ASI Series B* **294,** 21–107 (1992).

[31] Holst, T., Esteve, D., Urbina C. & Devoret, M. H. Effect of a Transmission Line Resonator on a Small Capacitance Tunnel Junction. *Phys. Rev. Lett.* **73,** 3455 (1994).

[32] Ingold, G.-L., Grabert, H. & Eberhardt, U. Cooper-pair current through ultrasmall Josephson junctions. *Phys. Rev. B* **50,** 395 (1994).

[33] Lu, W., Maranowski, K. D. & Rimberg, A. J. Charge transport processes in a superconducting single-electron transistor coupled to a microstrip transmission line. *Phys. Rev. B* **65,** 060501(R) (2002).

[34] Billangeon, P. -M. *et al.* Emission and Absorption Asymmetry in the Quantum Noise of a Josephson Junction. *Phys. Rev. Lett.* **96,** 136804 (2006).

[35] Toppari, J. J., Kühn, T., Halvari, A. P., Kinnunen, J., Leskinen, M. & Paraoanu, G. S. Cooper-pair resonances and subgap Coulomb blockade in a superconducting single-electron transistor. *Phys. Rev. B* **76,** 172505 (2007).

[36] Billangeon, P.-M., Pierre, F., Bouchiat H. & Deblock, R. ac Josephson Effect and Resonant Cooper Pair Tunneling Emission of a Single Cooper Pair Transistor. *Phys. Rev. Lett.* **98,**

216802 (2007).

[37] Pekola, J. P., Maisi, V. F., Kafanov, S., Chekurov, N., Kemppinen, A., Pashkin, Y. A., Saira, O.-P., Möttönen, M. & Tsai, J. S. Environment-Assisted Tunneling as an Origin of the Dynes Density of States. *Phys. Rev. Lett.* **105,** 026803 (2010).

[38] Pashkin, Yu. A., Im, H., Leppäkangas, J., Li, T. F., Astafiev, O., Abdumalikov, A. A., Thuneberg, Jr., E. & Tsai, J. S. Charge transport through ultrasmall single and double Josephson junctions coupled to resonant modes of the electromagnetic environment. *Phys. Rev. B,* **83,** 020502(R) (2011).

[39] Gabelli, J. *et al.* Electron-photon correlations and the third moment of quantum noise. *New J. Phys.* **15** 113045 (2013).

[40] Zakka-Bajjani, E., Dufouleur, J., Coulombel, N., Roche, P., Glattli, D. C. & Portier, F. Experimental Determination of the Statistics of Photons Emitted by a Tunnel Junction. *Phys. Rev. Lett.* **104,** 206802 (2010).

[41] Hofheinz, M., Portier, F., Baudouin, Q., Joyez, P., Vion, D., Bertet, P., Roche, P. & Esteve, D. Bright Side of the Coulomb Blockade. *Phys. Rev. Lett.* **106,** 217005 (2011).

[42] Bruhat, L. E., Viennot, J. J., Dartiailh, M. C., Desjardins, M. M., Kontos, T. & Cottet, A. Cavity Photons as a Probe for Charge Relaxation Resistance and Photon Emission in a Quantum Dot Coupled to Normal and Superconducting Continua. *Phys. Rev. X* **6,** 021014 (2016).

[43] Stockklauser, A., Maisi, V. F., Basset, J., Cujia, K., Reichl, C., Wegscheider, W., Ihn, T., Wallraff, A. & Ensslin, K. Microwave Emission from Hybridized States in a Semiconductor Charge Qubit. *Phys. Rev. Lett.* **115,** 046802 (2015).

[44] Childress, L., Sørensen, A. S. & Lukin, M. D. Mesoscopic Cavity Quantum Electrodynamics with Quantum Dots. *Phys. Rev. A* **69,** 042302 (2004).

[45] Liu, Y.-Y., Petersson, K. D., Stehlik, J., Taylor, J. M. & Petta, J. R. Photon Emission from a Cavity-Coupled Double Quantum Dot. *Phys. Rev. Lett.* **113,** 036801 (2014).

[46] You, J. Q., Liu, Y. X., Sun, C. P. & Nori, F. Persistent Single-Photon production by Tunable On-chip Micromaser with a Superconducting Quantum Circuit. *Phys. Rev. B* **75,** 104516 (2007).

[47] Astafiev, O., Inomata, K., Niskanen, A. O., Yamamoto, T., Pashkin, Y. A., Nakamura, Y. & Tsai, J. S. Single Artificial-Atom Lasing. *Nature* **449,** 588–590 (2007).

[48] Hauss, J., Fedorov, A., Hutter, C., Shnirman, A. & Schön, G. Single-Qubit Lasing and Cooling at the Rabi Frequency. *Phys. Rev. Lett.* **100,** 037003 (2008).

[49] Grajcar, M., van der Ploeg, S. H. W., Izmalkov, A., Ilichev, H. G. M. E., Fedorov, A., Shnirman, A. & Schön, G. Sisyphus Cooling and Amplification by a Superconducting Qubit. *Nat. Phys.* **4,** 612–616 (2008).

[50] Leivo, M. M., Pekola, J. P. & Averin, D. V. Efficient Peltier Refrigeration by a Pair of Normal Metal/Insulator/Superconductor Junctions. *Appl. Phys. Lett.* **68,** 1996–1998 (1996).

[51] Silveri, M., Grabert, H., Masuda, S., Tan, K. Y. & Möttönen, M. arXiv:1706.07188 (2017).

[52] Suri, B. *et al.,* Nonlinear microwave photon occupancy of a driven resonator strongly coupled to a transmon qubit. *Phys. Rev. A* **92,** 063801 (2015).

[53] Gevorgian, S., Linnér, L. J. P. & Kollberg, E. L. CAD Models for Shielded Multilayered CPW. *IEEE Trans. Microw. Theory Techn.* **43,** 772–779 (1995).

[54] Göppl, M., Fragner, A., Baur, M.; Bianchetti, R., Filipp, S., Fink, J. M., Leek, P. J., Puebla, G., Steffen, L. & Wallraff. A. Coplanar Waveguide Resonators for Circuit Quantum Electrodynamics. *J. Appl. Phys.* **104,** 113904–113911 (2008).
